# Toward a Socio-Material Approach to Cognitive Empathy in Autistic Spectrum Disorder

**DOI:** 10.3389/fpsyg.2019.02965

**Published:** 2020-01-10

**Authors:** Antonella Marchetti, Laura Miraglia, Cinzia Di Dio

**Affiliations:** ^1^Research Unit on Theory of Mind, Department of Psychology, Università Cattolica del Sacro Cuore, Milan, Italy; ^2^Faculty of Education, Università Cattolica del Sacro Cuore, Milan, Italy

**Keywords:** Theory of Mind, cognitive empathy, ToM precursors, ASD, child-adult relationship

In everyday life, actions and decisions are defined by a continuous interplay between cognitive and affective dimensions (Milani and Gagliardi, 2013; Lombardi et al., 2017) where empathy plays a central role (Eisenberg and Strayer, 1987). Empathy underpins the synchronous social response to another person's mental state and behavior. It represents a particular experience because it is not a directly accessible state: in contrast to other psychological constructs, it is neither a conduct, nor is always evident through specific behavioral expressions (Bonino et al., 1998). Empathy is like a dance between two individuals whose steps move between cognition and affects. Within a socio-material approach to development, in this paper we argue that motivated shared goal-directed actions toward an object may effectively mediate child-adult relationship by acting on the precursors of Theory of Mind (ToM), a cognitive component of empathy.

Feshbach (1978) defined empathy the *philosopher's stone* of human relationships. In this sense, it represents one of the most important mechanisms that contributes to regulate social relationships and human communication. Its two-dimensional nature—affective and cognitive—has spurred the interest of many authors who have tried to describe its evolution and development. According to Hoffman (1984), and in line with Davis et al. (1994) account, empathy manifests itself from the first days of life, initially quite entirely on the affective level. During the first year of life, children display *motor mimicry* by answering to the emotions they are witnessing (e.g., by crying when another infant is crying). This process is plausibly mediated by activation of the mirror mechanism allowing motor simulation (for review, see Rizzolatti and Sinigaglia, 2008) and “altercentric” participation (Braten, 1998). In this phase, the emotional response is involuntary and undifferentiated *(Global empathy*, Hoffman, 1984). Going through *egocentric empathy* during the second year of life, children spontaneously offer a kind of help which they would find comforting themselves and, in this sense, empathy is egocentric, although forms of early reasoning about the other's desires was observed already in 18-month-olds (Repacholi and Gopnik, 1997; see also, Astington et al., 1988). *Empathy for another's feelings* at the age of 3 years involves the development of role-taking skills, and children become aware that other people's feelings can differ from their own. The development of Theory of Mind (ToM), and namely the ability to conceptualize one's own and others' mental states underlying behavior (Wimmer and Perner, 1983) and social competence (Premack and Woodruff, 1978; Wellman et al., 2001), marks this maturation period. Children's responses to distress might become more appropriate to the other person's needs. We can now speak of empathy in its full meaning indicating that the child has developed the cognitive prerequisites enabling her/him to understand the other as a distinct person from her/himself (Bonino et al., 1998).

## Empathy in Atypical Development

Brain-imaging studies suggest that different but interacting brain structures are involved in cognitive and affective empathy (Shamay-Tsoory et al., 2009; Kalbe et al., 2010). These studies hypothesize that each individual component of the construct “empathy” can be selectively compromised with consequent specificities in behavioral impairments.

The Autistic Spectrum Disorder (ASD) is featured by two main types of impairment affecting social and language competencies, on the one hand, and involving stereotypical and repetitive behaviors, on the other (American Psychiatric Association, 2013, APA). One of the central issues is if and how ASD individuals empathize. Eye tracking experiments (Frith, 2003) have shown that autistic individuals, when looking for the meaning of a dynamic social situation, do not direct attention to very expressive aspects of the image. For example, they attend more to the peripheral area of the face instead of the eyes and mouth to which particular attention is typically paid (e.g., Savazzi et al., 2014; Di Dio et al., 2019). Furthermore, ASD individuals' *mind-blindness* (Baron-Cohen, 1995) leads to the inability to attribute mental states and to an inadequate conception of feelings (Baron-Cohen et al., 2001; Frith, 2003; Fabio et al., 2011). ASD individuals are generally unable to recognize and name emotions, read expressive cues and take the others' point of view and perspective, reasons that may—at least partly—explain ASD general inability to provide appropriate emotional responses. Not only mentalization skills allow people to understand the other's emotions, but importantly the reasons underlying them, thus making it is possible to respond appropriately to the other person's state of mind. If we see a sad person, it is thanks to our mentalization skills that we can understand if s/he needs relief or prefers staying alone. Nevertheless, ASD individuals are not indifferent to the other's suffering. They are able to *instinctive sympathy* (Frith, 2003), i.e., to involuntary respond to basic emotional reactions, although their inability to readily and coherently attribute a mental state will most likely lead to a socio-behavioral failure, with possible consequent experience of depression and anxiety (Conti et al., 2015).

## Can a Socio-material Approach Promote Empathy?

Socio-materiality is clearly the fusion between the terms “social” and “material,” and it has the potential to link materiality to each and every phenomenon that we consider social (Leonardi et al., 2012). The socio-material theory assumes that human activity is mediated by tools (Leonardi, 2012). Actions supported by tool-use and intentionally aimed at production create thought (Vygotsky, 1978).

The conceptual underlie of our argumentation resides in the acknowledged nature of the dyadic relationship that commonly develops between the child and the adult in early childhood. In typical development, the dyadic relationship generally invites the triadic relationship by including the use of an object in its (culturally determined) typical function (Leontiev, 1981; Costall, 1997; Rodríguez and Moro, 2008; Barthélémy-Musso et al., 2013). Adults and children readily construct action representations organized with respect to an ultimate goal, allowing one to predict the consequences of action, interpret and describe actions, and categorize action sequences (Sommerville et al., 2005). Already 1-year-old infants possess a genuine understanding of other persons as intentional and attentional agents (Tomasello and Haberl, 2003). In a compromised dyadic relationship, where the infant fails or struggles to include the other in her/his action zone and mental sphere, the object may become the primary referent of the dyadic relationship. The child concentrates on the relationship with the object most possibly because s/he may ignore the emotional and mental pressures that characterize the typical relationship with the adult (Lecciso et al., 2013). The socio-material perspective suggests that through the initiation of a dyadic relationship with the object, it is possible to include the adult in the triadic relationship. So, from a typical child-adult-object interaction, the relationship shifts to the child-object-adult interaction. That objects can become the mediators of a compromised adult-child social interactions associated with attentional deficits has been already theorized (Rodríguez and Moro, 2008; Sinha and Rodríguez, 2008; Sinha, 2015). The human psychological structure is modeled and transformed by acting in the world and manipulating objects. Gradually, as the physical/sensori-motor representation of the world—which also includes the relation with the adult (see, Braten, 2006)—the child builds a representation of the mental self and the mental other. This development is not abrupt, but moves from a concrete stage to a representational stage also thanks to the developmental of processes and behaviors recognized as the precursors of ToM.

Before the acquisition of false belief at about 4 years of age, the child progressively builds an understanding of the mind. In typical development, the dyadic relationship (classically affective in nature) with the caregiver opens the child up to a triadic relationship with the world through the precursors of ToM that develop within the first 2 years of life: joint-attention, pointing (indicative), performative (from requesting to declarative), understanding of agency, pretend play. In a compromised child-adult relationship, object may possibly bring the child closer to the other during *shared goal-directed actions* on an object. Under this condition, the child-adult's responses are contingent on a common object of interest (motivation and openness). When the other responds to the stimulus in the same way as does the child, an initial “like me” relational form may develop which starts from the child's experience of the other's objective/concrete and sensori-motor characteristics. Subsequently, the child may begin to form an association between the self and the other that includes subjective characteristics (both subjects are the same and different from the object) that are discovered through doing. Then, by intervening on the precursors of ToM, and in particular on joint attention, referential communication, and motivation, the other (and her/his mind) may be gradually included as a referential agent in a triadic relationship. A differentiation is initiated which potentially leads the child to the understanding of the other as an individual with a mind that may be different from her/his own.

The observational work by Iannaccone et al. (2018) right supports this idea. The authors preliminarily showed that objects may serve as concrete mediators in the intersubjective space between adult and ASD infants aged between 18 and 24 months during object manipulation and building a tower of toy blocks. Additionally, some of the infants' attention patterns were visibly mediated by the object, in that the children monitored the adult's attention through eye contact or by restarting manipulation of the blocks, a process labeled “object-mediated attention.” In clinical settings, the practice of including objects during therapy is already widely used as a means to establishing a connection with the patient. In this respect, Conti et al. (2015) suggest that—by generating a high degree of motivation and engagement in the child (Scassellati, 2002)—the use of robots can be effectively integrated in current ASD therapies by developing protocols aimed to implement, for example, imitation skills, which are basic to the development of social competencies. The object “robot” becomes a mediator of the child-adult relationship. In principal, by working on ToM precursors through mediation of object-use, mind understanding and social-communicative competencies may be promoted in ASD individuals with and without intellectual disability: what matters is, in fact, the scaffolder's ability to properly place her/himself within the child's zone of proximal development to enhance her/his abilities at any level of intellectual functioning (see, Conti et al., 2018; see also, Fabio et al., 2013).

Concluding, the development of the grasp of the other's mind in terms of emotions, intentions, desires and—then—beliefs and false beliefs at increasing levels of cognitive complexity is important because, with it, the child begins to reason in terms of subjectively founded “true and false” and no longer in terms of objective “true and false.” To understand that the mind represents the world and that mental representations guide action is the crucial step for the acquisition of ToM. Theory of Mind involves a representation of the subjectivity of one's own and the other's mental states at a high degree of interindividual variability. When the dyadic component is impaired, by having the child establishing a relationship with the material object, the object can, from a socio-material perspective, open the child up to a triadic relationship (child-object-adult). This, in turn, may help the “motivated” child enter the dyadic child-adult relationship in a backward path, thus allowing the recovery and implementation of—at least some—precursors of the ToM competence. The meaning of the objects helps the individual build a meaning of the person (mind). Interventions have been developed worldwide to improve ToM skills of individuals with autism. Despite these efforts, little is known about whether, when, where and for whom these treatment programs work in autism (Begeer et al., 2011). We believe that it could be helpful to look into methods of intervention which embrace a socio-material perspective allowing to promote empathic skills by starting from its basic relational components. Our suggestion to intervene timely on ToM precursors is in line with general emphasis on early diagnosis. According to Bruner (1986), before children have acquired the “certificate” of false belief, adults and children had made a very long journey toward each other's mind. And, surely, it is in Bruner's vigoskijan spirit that this journey is populated with objects.

## Author Contributions

All authors listed have made a substantial, direct and intellectual contribution to the work, and approved it for publication.

### Conflict of Interest

The authors declare that the research was conducted in the absence of any commercial or financial relationships that could be construed as a potential conflict of interest.
